# Factors potentially associated with the decision of admission to the intensive care unit in a middle-income country: a survey of Brazilian physicians

**DOI:** 10.5935/0103-507X.20170025

**Published:** 2017

**Authors:** João Gabriel Rosa Ramos, Rogerio da Hora Passos, Paulo Benigno Pena Baptista, Daniel Neves Forte

**Affiliations:** 1 Programa de Pós-Graduação em Ciências Médicas, Faculdade de Medicina, Universidade de São Paulo - São Paulo (SP), Brasil.; 2 Unidade de Terapia Intensiva, Hospital São Rafael - Salvador (BA), Brasil.; 3 Faculdade de Medicina, UNIME - Lauro de Freitas (BA), Brasil.; 4 Unidade de Terapia Intensiva, Hospital Português - Salvador (BA), Brasil.; 5 Unidade de Terapia Intensiva, Disciplina de Emergências Clínicas, Hospital das Clínicas, Faculdade de Medicina, Universidade de São Paulo - São Paulo (SP), Brasil.; 6 Equipe de Cuidados Paliativos, Hospital Sírio-Libanês - São Paulo (SP), Brasil.

**Keywords:** Critical care, Decision making, Resource allocation, Intensive care units

## Abstract

**Objective:**

To evaluate the factors potentially associated with the decision of admission to the intensive care unit in Brazil.

**Methods:**

An electronic survey of Brazilian physicians working in intensive care units. Fourteen variables that were potentially associated with the decision of admission to the intensive care unit were rated as important (from 1 to 5) by the respondents and were later grouped as "patient-related," "scarcity-related" and "administrative-related" factors. The workplace and physician characteristics were evaluated for correlation with the factor ratings.

**Results:**

During the study period, 125 physicians completed the survey. The scores on patient-related factors were rated higher on their potential to affect decisions than scarcity-related or administrative-related factors, with a mean ± SD of 3.42 ± 0.7, 2.75 ± 0.7 and 2.87 ± 0.7, respectively (p < 0.001). The patient's underlying illness prognosis was rated by 64.5% of the physicians as always or frequently affecting decisions, followed by acute illness prognosis (57%), number of intensive care unit beds available (56%) and patient's wishes (53%). After controlling for confounders, receiving specific training on intensive care unit triage was associated with higher ratings of the patient-related factors and scarcity-related factors, while working in a public intensive care unit (as opposed to a private intensive care unit) was associated with higher ratings of the scarcity-related factors.

**Conclusions:**

Patient-related factors were more frequently rated as potentially affecting intensive care unit admission decisions than scarcity-related or administrative-related factors. Physician and workplace characteristics were associated with different factor ratings.

## Abstract (pt)

**Objetivo:**

Avaliar os fatores potencialmente associados à decisão de admitir um paciente à unidade de terapia intensiva no Brasil.

**Métodos:**

Foi realizado um levantamento eletrônico de médicos brasileiros atuantes em unidades de terapia intensiva. Catorze variáveis consideradas potencialmente associadas à decisão de admitir um paciente à unidade de terapia intensiva foram pontuadas como importante (de 1 a 5) pelos participantes e, mais tarde, agrupadas como fatores "relacionados ao paciente", "relacionados à escassez" e "relacionados à administração". O ambiente de trabalho e as características do médico foram avaliados quanto à sua correlação com as pontuações dos fatores.

**Resultados:**

Durante o período do estudo, 125 médicos preencheram o formulário. Os escores dos fatores relacionados ao paciente foram pontuados, em termos de seu potencial para afetar as decisões, em um nível mais alto do que os fatores relacionados à escassez ou à administração, com média (mais ou menos o desvio padrão), respectivamente, de 3,42 ± 0,7, 2,75 ± 0,7 e 2,87 ± 0,7 (p < 0,001). O prognóstico da doença de base do paciente foi classificado em 64,5% pelos médicos como afetando sempre ou frequentemente as decisões, seguido por prognóstico da doença aguda (57%), número de leitos disponíveis na unidade de terapia intensiva (56%) e vontade dos pacientes (53%). Após o ajuste de fatores de confusão, o recebimento de treinamento específico em triagem para terapia intensiva se associou com escores mais elevados dos fatores relacionados ao paciente e à escassez, enquanto o fato de trabalhar em uma unidade de terapia intensiva pública (em oposição a trabalhar em uma unidade de terapia intensiva privada) se associou com gradações mais elevadas para fatores relacionados à escassez.

**Conclusões:**

Os fatores relacionados ao paciente foram classificados como tendo potencial de afetar as decisões de admissão à unidade de terapia intensiva mais frequentemente do que fatores relacionados à escassez ou à administração. As características do médico e do ambiente de trabalho se associaram com classificações diferenciais dos fatores.

## INTRODUCTION

Decisions on admission to the intensive care unit (ICU) are performed routinely worldwide.^(1)^ These decisions may be associated with the patients' clinical characteristics,^(2-4)^ but they are also influenced by non-clinical factors, such as ICU bed availability.^(2,4,5)^

Despite the development of guidelines, there is no international consensus on how to manage these triage decisions.^(6-8)^ Moreover, the triage processes seem to be contextually and culturally sensitive, with great variability between different countries^(5,9,10)^ and, even, within countries.^(11)^ Specifically, in low-income and middle-income countries, in which resources are more scarce,^(12-14)^ such triage decisions may be more heavily influenced by non-patient related factors.^(15)^ However, in Brazil and other low-income and middle-income countries, there is paucity of data regarding these allocations decisions.^(16-19)^

In this study, we aimed to analyze the factors that potentially influence Brazilian physicians' decisions of admission to the intensive care unit and to examine the association of specific physician and workplace characteristics with these factors.

## METHODS

This study was approved by the *Hospital das Clínicas* of the *Faculdade de Medicina* of the *Universidade de São Paulo* (USP) institutional review board (approval number 1.015.441), which approved the utilization of an electronically obtained informed consent.

This study was an electronic, self-administered survey. An online questionnaire was developed and made available electronically (SurveyMonkey Inc., USA).

A convenience sample of Brazilian physicians with experience in critical care was invited to participate in the study by medical specialty e-mail groups, social media networking and personal contacts. Invitations were sent on three different occasions, within two-week intervals, initiating in October 2015.

Respondents were included if they were licensed practicing physicians who were currently working in ICUs. Participants were excluded if consent was not obtained or if the research questionnaire was not fully completed. Participants were not required to be board-certified in critical care to participate in the study.

The online survey included the demographic and professional characteristics of the respondents and their ICU, such as whether there was high-intensity staffing (defined as the presence of a critical care specialist at least 4 hours per day, at least 5 days per week). The survey included variables related to the respondents' exposure to situations of ICU bed scarcity and triage in their practice (i.e., if the physicians were exposed to situations of ICU bed scarcity and if the physician is involved in the ICU triage process). Other characteristics were also collected: gender, age, state and city of current practice, age, date of graduation from medical school, fellowship in intensive care medicine (i.e., board-eligible in intensive care medicine), board certification in intensive care medicine, other medical specialization, number of hours per week working in ICU, working in public or private ICU, "closed" or "open" ICU and number of beds in the ICU. If the physician worked in more than one ICU, it was asked that the responses reflect the ICU in which the physician worked most of the time.

To assess the importance of factors in the decisions of admission to the ICU, the respondents were asked to rate 14 factors as being potentially important to decision-making, using a Likert scale from 1 (never affects decisions) to 5 (always affects decisions). Later, those factors were gathered into three groups: (1) patient-related factors (comprising patient's age, underlying illness prognosis, previous performance status, acute illness prognosis, patient's wishes, relatives' wishes); (2) scarcity-related factors (comprising number of ICU beds available, full operating room, current ICU workload, probable ICU admission cost) and (3) administrative-related factors (comprising institutional admission policy, pressure from the requesting physician, pressure from the family, fear of malpractice suits).

### Statistical analysis

Microsoft Excel 365^TM^ (Microsoft, USA) and Statistical Package for Social Sciences (SPSS), version 21.0 (SPSS Inc., USA) were utilized as database and statistical software, respectively.

Continuous data were described as the mean ± standard deviation and were analyzed by Student's *t*-test. Categorical variables were described as a number (percentage) and were analyzed by chi-squared or Fisher's exact test, when appropriate. The relevance of factors related to the decision of admission to the ICU, as measured by the Likert scale, was analyzed as a dichotomous variable (dichotomized as frequently or always affects decisions vs. sometimes, rarely or never affects decisions) or as an ordinal variable. The grouped variables (patient-related, scarcity-related and administrative-related factors) were calculated as the overall mean of the factors comprised in each group. A post hoc analysis evaluating the difference between physicians working in public or private ICUs was performed because it was thought to be of relevance.

The correlation of the respondent characteristics with the factors associated with the decision of admission to the ICU was evaluated by a Spearman's rank correlation test. All respondent characteristics that were associated with a p value < 0.1 were entered in a multivariable linear regression model to control for confounding. Collinearity was assessed using the tolerance test and variance inflation factor. Three different models were built, one for each group of variables (patient-related, scarcity-related and administrative-related factors). A two-tailed p value of 0.05 was considered significant in all analyses.

## RESULTS

In the study period, 178 physicians logged on to the questionnaire and 125 (70.2%) complete responses were collected and analyzed. The respondent characteristics are depicted in table 1. There were respondents from all Brazilian regions and 15 different Brazilian states. Most respondents (81; 65%) were from the Southeast region; followed by the Northeast region (24; 19%); South region (9; 7%); Midwest region (8; 6%) and North region (3; 2%).

**Table 1:** Characteristics of the respondents and their intensive care units (N = 125)

Characteristics	
Male sex	87 (70.2)
Age (years)	37 ± 7.4
Years since medical graduation	13 ± 7.9
Board-certified or board-eligible in critical care medicine	95 (76)
Hours per week working in ICU	
< 12	4 (3.2)
12 - 24	15 (12)
24 - 40	36 (28.8)
> 40	70 (56)
Closed ICU	77 (61.6)
Public ICU	58 (46.4)
High-staffed ICU	123 (98.4)
Number of ICU beds	22.4 ± 16.1
Rarely exposed to situations of ICU bed scarcity	38 (30.4)
Rarely involved in ICU triage process	52 (41.6)
Has received specific training on ICU triage	20 (16)

ICU - intensive care unit. The results are expressed as N (%) or mean ± standard deviation.

Most respondents (87; 70.2%) were male, mean age ± SD was 37 ± 7.4 years and 95 respondents (76%) were board-eligible or board-certified in critical care. Almost all respondents worked in high-staffed ICU, but close to half of the respondents worked in public ICU or closed ICU, as opposed to private or open ICU. Of the 48 respondents that worked in open ICU, most (39; 81.3%) worked in private ICU. Only 38 (30.4%) respondents were rarely exposed to situations of ICU bed scarcity and 52 (41.6%) were rarely involved in the ICU triage process (Table 1). The majority of respondents who were rarely exposed to situations of ICU bed scarcity (35; 92.1%) and were rarely involved in ICU triage (46; 88.5%) worked in private ICU.

### Factors associated with the decision of admission to the intensive care unit

**Figure 1:**
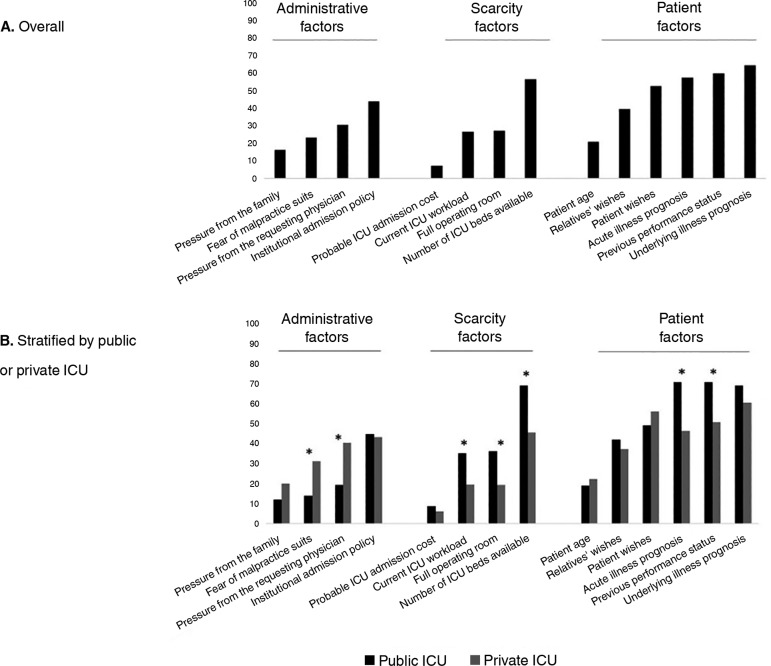
Respondents' ranking of factors that were judged to be important or very important for the overall decision of intensive care unit admission (A) and the rankings stratified by working in a public or private intensive care unit (B). ICU - intensive care unit. * p<0.05 between physicians working in public or private intensive care units.

Most respondents (80; 64.5%) rated the underlying illness prognosis as always or frequently affecting the decisions of admission to the ICU (Figure 1). Previous performance status, acute illness prognosis, patient's wishes and number of ICU beds available were also rated as always or frequently affecting these decisions by more than half of the respondents (Figure 1A).

There were significant differences between the physicians working in public or private ICU in the ratings of individual factors (Figure 1B). Physicians working in public ICU, when compared to those working in private ICU, were more likely to rate previous performance status, acute illness prognosis, number of ICU beds available and full operating room as important. Physicians working in private ICUs, when compared to those working in public ICU, were more likely to rate pressure from the requesting physician and fear of malpractice suits as important.

**Figure 2:**
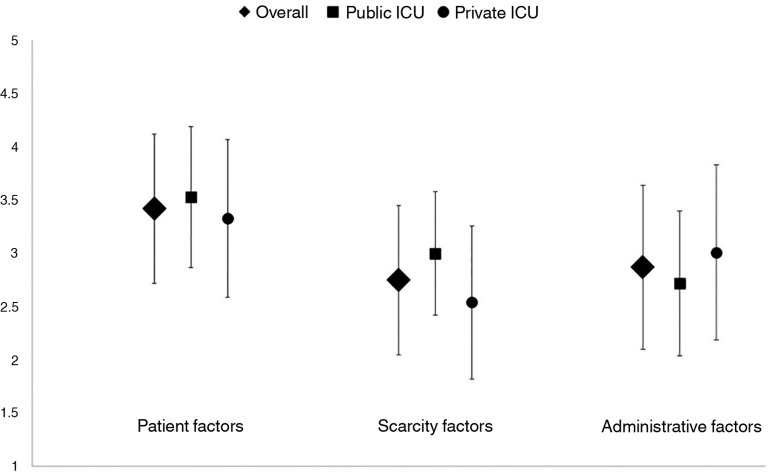
Mean (SD) ratings for each group of factors associated with the decision of intensive care unit admission, overall and stratified by working in public or private intensive care unit. See text for statistical tests. ICU - intensive care unit.

Overall, patient-related factors were given higher scores (in a range from 1 to 5) than scarcity-related factors and administrative-related factors, with a mean ± SD of 3.42 ± 0.7, 2.75 ± 0.7 and 2.87 ± 0.7, and p < 0.001 for the comparison between patient-related factors and scarcity- or administrative-related factors and p = 0.12 for the comparison between scarcity-related factors and administrative-related factors (Figure 2).

When comparing physicians working in public or private ICU (Figure 2), scarcity-related factors were rated higher in public ICU than in private ICU (3.0 ± 0.58 and 2.54 ± 0.72, respectively, p < 0.001) and administrative-related factors were rated lower in public ICU than in private ICU (2.72 ± 0.68 and 3.01 ± 0.82, respectively, p = 0.037). There was no significant difference in patient-related factors between public ICU (3.53 ± 0.66) and private ICU (3.33 ± 0.74), p = 0.138.

### Respondent characteristics and factors associated with the decision of intensive care unit admission

Different respondent characteristics were associated with patient-related factors, scarcity-related factors or administrative-related factors (Table 2). After adjusting for confounders (Table 3), receiving specific training on ICU triage was associated with a higher rating of patient-related factors, while working in a public ICU and specific training on ICU triage were associated with a higher rating of scarcity-related factors. No specific characteristics were associated with administrative-related factors.

**Table 2:** Correlation of respondent characteristics with factors associated with the decision of intensive care unit admission

Respondent characteristics	Patient factors		Scarcity factors		Administrative factors	
	Spearman coefficient	p value	Spearman coefficient	p value	Spearman coefficient	p value
Male sex	0.022	0.815	0.191*	0.037	0.028	0.764
Age	-0.042	0.939	-0.061	0.506	-0.019	0.834
Years since graduation	-0.007	0.939	-0.052	0.570	-0.017	0.857
Board certification or eligibility in critical care	0.152†	0.097	0.080	0.383	-0.076	0.407
Hours per week working in the ICU	0.089	0.330	0.049	0.592	-0.044	0.632
"Open" versus "Closed" ICU	-0.156†	0.088	-0.216*	0.017	0.208*	0.022
Private versus public ICU	-0.113	0.219	-0.320*	<0.001	0.180*	0.048
High-staffed ICU	-0.079	0.388	-0.033	0.722	0.055	0.549
Number of ICU beds	0.021	0.815	-0.081	0.377	0.077	0.403
Specific training on ICU triage	0.161†	0.077	0.187*	0.040	0.041	0.658
Rarely exposed to ICU bed scarcity	0.060	0.515	-0.171†	0.061	0.114	0.213
Rarely involved in ICU triage	0.016	0.863	-0.174†	0.056	0.202*	0.027

ICU - intensive care unit.

† p < 0.10;

* p < 0.05.

**Table 3:** Multivariate linear regression analyses for the association of respondent characteristics with the factors associated with the decision of ICU admission. Grouped as (A) patient-related factors; (B) scarcity-related factors and (C) administrative-related factors

Characteristic	B coefficient	95%CI		p value
		Lower	Upper	
(A) Patient-related factors				
Board certification or eligibility in critical care	0.220	-0.072	0.511	0.138
"Open" versus "closed" ICU	-0.232	-0.490	0.025	0.077
Specific training on ICU triage	0.359	0.026	0.692	0.035
R^2^ = 0.079; p = 0.022.				
(B) Scarcity-related factors				
Male sex	0.199	-0.062	0.460	0.133
"Open" versus "closed" ICU	-0.196	-0.482	0.089	0.176
Private versus public ICU	-0.328	-0.652	-0.004	0.047
Specific training on ICU triage	0.350	0.015	0.685	0.040
Rarely exposed to ICU bed scarcity	0.000	-0.310	0.309	0.998
Rarely involved in ICU triage	0.011	-0.304	0.326	0.945
R^2^ = 0.167; p = 0.002.				
(C) Administrative-related factors				
"Open" versus "closed" ICU	0.183	-0.140	0.505	0.265
Private versus public ICU	0.134	-0.233	0.501	0.472
Rarely involved in ICU triage	0.122	-0.238	0.483	0.503
R^2^ = 0.053; p = 0.095.				

95%CI - 95% confidence interval; ICU - intensive care unit. Lower - lower limit of the 95% confidence interval. Upper - upper limit of the 95% confidence interval.

## DISCUSSION

In this study of factors potentially affecting Brazilian physicians' decisions of admission to the ICU, we have found that patient-related factors were mostly regarded as always or frequently affecting these decisions. However, scarcity-related factors, such as ICU bed availability, were also rated as highly influencing these decisions. Receiving specific training in ICU triage was associated with higher ratings in patient-related factors, while receiving specific training in ICU triage and working in a public ICU (as opposed to a private ICU) were associated with higher ratings in scarcity-related factors.

This survey demonstrated that patient-related factors were more frequently rated as potentially affecting decisions on admission to the ICU. Other studies performed in Switzerland^(5)^ and in the Netherlands^(10)^ demonstrated similar results. However, one study evaluating physicians from the United States (US) and Israel demonstrated different patterns,^(9)^ with less US physicians rating ICU admission decision details regarding patient-related factors as important.

Most respondents in the survey indicated that they had experienced situations of ICU bed scarcity; however, 41% were rarely or never involved in the ICU triage process. Because the respondents who rarely experienced situations of ICU bed scarcity and were rarely involved in the ICU triage process usually worked in private ICU, this might reflect the different resource availabilities and ICU workflows in public and private ICU. This result is further supported by the fact that most respondents indicated that private ICU were functioning as open ICUs.

In a *post hoc* analysis, we found significant differences between the physicians working in public and private ICU. Overall, scarcity-related factors were deemed as more important by physicians working in public ICU, while administrative-related factors were deemed as more important by physicians working in private ICU. Nevertheless, patient-related factors were deemed as more important than scarcity-related or administrative-related factors for both physicians working in public and those in private ICU, even though there were differences in the ratings of specific factors, such as the influence of acute illness prognosis and previous performance status on the decision of admission to the ICU. It is possible that these rating differences reflect actual differences in practice, contributing to inequalities in the delivery of care. Whether such differences are driven exclusively by resource availability or if other factors, such as different workflow designs and different financial incentives, are contributory remains elusive.

We found that some respondent characteristics were associated with higher ratings of patient-related factors and scarcity-related factors as potentially affecting decisions on ICU admission. Specific training on ICU triage was associated with higher ratings of patient-related factors and scarcity-related factors. Although not specified, training in ICU triage may be a modifiable factor in enhancing triage processes. Working in a private ICU was associated with a lower rating of scarcity-related factors as potentially affecting decisions on ICU admission. Although this association was adjusted for respondents who were rarely exposed to situations of ICU scarcity and were rarely involved in ICU triage, this result most likely represents a difference in resource availability in daily practice. Of note, although statistically significant, these associations were weak and should be interpreted with caution.

This study utilized a web-based survey, which may have advantages, such as a higher accuracy of responses and a lower risk of errors from its limited manual data entry, but it also exhibits variable responses rates.^(10)^ We could not calculate the exact response rate because we did not have a specific denominator, but we had a high completion rate. Moreover, this study was based on a convenience, non-aleatory sample, which further increases the risk of bias. Despite this limitation, there were responses from all five regions and from 15 of the 27 states in Brazil and, aside from an overrepresentation of the Northeast region, the distribution of respondents followed the distribution of intensivists among the different regions of Brazil.^(20)^ Respondents were younger than the average intensivist in Brazil, but they had the same gender distribution, as it has been demonstrated that the mean age of intensivists in Brazil is 47.5 ± 9.5 and 69.8% are male.^(20)^ However, these data^(20)^ reflect the distribution of physicians certified in intensive care medicine, not of all the physicians working in ICU. In our study, there was a high rate of physicians who were board-eligible or board-certified in intensive care medicine, which probably does not reflect the current reality in most ICU in Brazil, in which most physicians working in the ICU are not intensive care specialists. Intensive care specialists will probably have different attitudes toward ICU admission than non-specialists, which may bias our results. Nonetheless, although it is not required to be an intensive care specialist to work in an ICU, it is required that all ICU have a medical director who must be board-certified in intensive care medicine. Because ICU directors are usually involved in developing ICU admission policies and guidelines, in addition to helping in difficult cases, it is possible that our results are generalizable.

Additionally, Brazil has a unique health system in that, although there is a Unified Health System^(21)^ that is government-led with no direct payment from users, there is also a private sector that covers a smaller proportion of the population.^(22)^ These characteristics have led to inequalities that are translated into the availability of healthcare resources, including ICU.^(14)^ However, although these characteristics are unique to Brazil, other countries face similar challenges.^(23)^ Finally, this survey intended to measure the factors that potentially affect decisions on admission to the ICU in a hypothetical manner. It is possible that actual behaviors may differ from what is depicted in a survey, which could also bias the results.

## CONCLUSION

In this survey of Brazilian physicians working in intensive care units, patient-related factors were more frequently rated as potentially affecting intensive care unit admission decisions than scarcity-related or administrative-related factors, even though intensive care unit bed availability was also an important factor. Specific training on intensive care unit triage was associated with higher ratings of patient-related factors and scarcity-related factors, while working in a public intensive care unit (as opposed to private intensive care unit) was associated with higher ratings of scarcity-related factors.
